# Ecotoxicity and fate of silver nanomaterial in an outdoor lysimeter study after twofold application by sewage sludge

**DOI:** 10.1007/s10646-022-02529-3

**Published:** 2022-03-09

**Authors:** Martin Hoppe, Jan Köser, Kerstin Hund-Rinke, Karsten Schlich

**Affiliations:** 1 Federal Institute for Geosciences and Natural Resources, Hanover, Germany; 2 Fraunhofer Institute for Molecular Biology and Applied Ecology, Schmallenberg, Germany

**Keywords:** Silver nanomaterial, Silver nitrate, Plant uptake, Long-term experiment, Ammonium oxidizing bacteria, Soil

## Abstract

The increasing use of antibacterial silver nanomaterials (AgNM) in consumer products leads to their release into sewers. High amounts of AgNM become retained in sewage sludge, which causes their accumulation in agricultural soils when sewage sludge is applied as fertilizer. This increase in AgNM arouses concerns about toxicity to soil organisms and transfer within trophic levels. Long-term field studies simulating the sewage sludge pathway to soils are sparse, and the effects of a second sewage sludge application are unknown. In this perennial field lysimeter study, a twofold application of AgNM (NM-300K, 2 + 3 mg AgNM/kg dry matter soil (DMS)) and a onefold application of silver nitrate (AgNO_3_, 2 mg Ag/kg DMS) by sewage sludge to the uppermost 20 cm of the soil (Cambisol) were applied. The response of microorganisms to the applications was determined by measuring the inhibition of ammonium-oxidizing bacteria (AOB). Silver concentration in soil, leachates, and crops were measured after acid digestion by inductively coupled plasma mass spectrometry (ICP-MS). Almost no vertical Ag translocation to deeper soil layers and negligible Ag release to leachates suggest that soil is a large sink for AgNM and AgNO_3_. For AgNM, an increase in toxicity to AOB was shown after the second sewage sludge application. The application of AgNO_3_ resulted in long-term toxicity comparable to the toxicity of AgNM. Low root uptake from both AgNM- and AgNO_3_-spiked lysimeters to crops indicates their incomplete immobilization, which is why food chain uptake cannot completely be excluded. However, the root-shoot barrier for wheat (9.8 → 0.1 mg/kg) and skin body barrier for sugar beets (1.0 → 0.2 mg/kg) will further reduce the accumulation within trophic levels. Moreover, the applied AgNM concentration was above the predicted environmental concentration, which is why the root uptake might be negligible in agricultural practice.

## Highlights

- In a perennial field study, most of the applied AgNM and AgNO_3_ remained immobile in soil.
- The release of Ag to the leachates was very low (<176 ng/L).
- Low, continuous, species-specific transfer of AgNM and AgNO_3_ from soil to wheat and sugar beet.
- Constant and comparable inhibition of AOB by AgNM and AgNO_3_.
- Second sewage sludge application (AgNM) after four years leads to increased toxicity.

## Introduction

Silver nanomaterials are one of the most widely used nanomaterials (NM) in various consumer products because of their antimicrobial properties (Benn et al. 2010). The production and use of AgNM-containing products lead to increased AgNM emissions into wastewater (Bundschuh et al. 2018). Thus, the entry of AgNM into wastewater treatment plants (WWTPs) is inevitable (Kaegi et al. 2013). High amounts of AgNM become retained in sewage sludge (Westerhoff and Nowack 2013; Schlich et al. 2013). Therefore, the transfer of sewage sludge to agricultural soils is one of the most important NM pathways into the environment (Tourinho et al. 2012; Pan and Xing 2012). Several short-term studies showed a rather high retention of AgNM for most agricultural soils, which is why soils were considered a large sink for AgNM (Pan and Xing 2012; Cornelis et al. 2014; Hoppe et al. 2014, 2015; Wang et al. 2018).

The accumulation of potentially toxic AgNM in soil leads to concerns about the soil bacterial community (Forstner et al. 2020). Ecotoxicological experiments with AgNM under standardized conditions confirmed that pure AgNM affects the biomass content by substrate-induced respiration, the enzyme activity of soils and ammonia oxidizing bacteria (Hänsch and Emmerling 2010; Schlich and Hund–Rinke 2015). Partial sulfidation of AgNM was found in experiments with raw sewage sludge (Kaegi et al. 2013). This process might decrease the toxicity of AgNM and potentially limit their environmental impact (Levard et al. 2012). Otherwise, AgNM introduced to a model sewage treatment plant, applied to soil via sewage sludge and tested after 100–140 d, remained as toxic toward soil microorganisms as freshly prepared AgNM applied to soil and tested after 28 d (Schlich et al. 2013). This was also demonstrated in studies in which AgNM was first sulfidized (Kraas et al. 2017; Schlich et al. 2018). In addition, Forstner et al. (2020) showed for a test duration of 90 d that the effects of processed AgNM on soil bacterial communities were larger and more persistent than those of fresh AgNM, silver sulfide nanomaterial (Ag_2_S-NM), and AgNO_3_. In general, Spurgeon et al. (2020) addressed the urgent need for long-term studies about the toxicity of NM.

Metal-based NM can cross the root barrier and translocate through the vascular system into various tissues, which is controlled by NM physicochemical characteristics, plant species and the rate of transpiration (Ruotolo et al. 2018). Schlich et al. (2018) applied different NM (AgNM, Ag_2_S-NM) and AgNO_3_ to soils by sewage sludge and obtained Ag uptake to oat (*Avena sativa* L.), whereby the shoots contained less Ag than the roots. In general, the accumulation of AgNM in soil leads to major concern about the food chain due to modifications in the nutrient values of food crops and transfer within trophic levels (Gardea–Torresdey et al. 2014). However, long-term studies that elucidate the fate and toxicity of AgNM and silver ions (Ag^+^) under realistic environmental conditions are still very sparse (Schlich et al. 2017). Yang et al. (2020) investigated the fate of biosolid-borne Ag in an outdoor field study and found Ag accumulation in the soil and a very low Ag (<1‰) uptake to crops. Outdoor lysimeter experiments confirmed that AgNM was associated with soil colloids and showed a very low breakthrough (<0.1%) to undisturbed loamy sandy soil (Makselon et al. 2018). Low release of Ag from AgNM-spiked sandy soil to the leachates of a field lysimeter was also found in our previous study, which also exhibited toxicity to AOB and Ag root uptake to wheat and canola (Schlich et al. 2017). Despite the fact that knowledge about the fate and impact of AgNM in soil has constantly grown in the last decade, Courtois et al. (2019) reviewed that much remains unclear about the ecotoxicology of Ag species in soil, especially with respect to a possible entry to the trophic food chain by accumulation in plant tissues. In particular, AgNM root uptake to agricultural crops such as wheat and canola (Schlich et al. 2017) raised concern about AgNM uptake to sugar beets, which might pave the pathway into the human food chain. Moreover, the AgNM fate and toxicity after repetitive AgNM-containing sludge application to agricultural soil under field conditions are still unknown.

This long-term study was conceptualized to address these knowledge gaps about AgNM fate and toxicity after the second application (first application 2014, second application 2018) of AgNM-spiked and unspiked sewage sludge to soil. In addition, the study also aimed to investigate the fate and effect of AgNO_3_ as an ionic Ag source in the field lysimeter experiment, which was not part of the first study published in 2017 (Schlich et al. 2017). This study investigates (i) the vertical translocation of AgNM and AgNO_3_ in the uppermost 40 cm of the lysimeter, (ii) the release of Ag to the leachates, (iii) the uptake of Ag to wheat roots and sugar beets, and (iv) the inhibition of AOB.

Therefore, soil samples were digested with aqua regia, plant samples were digested in nitric acid (HNO_3_), and water samples were filtered and acidified with HNO_3_. Afterward, the Ag concentration in the samples was measured with inductively coupled plasma optical emission spectroscopy (ICP-OES) and inductively coupled plasma mass spectrometry (ICP-MS). Following ISO 15685 (2012), the inhibition of AOB was measured to determine the response of microorganisms to the application of AgNM and AgNO_3_ to the soil (Cambisol).

## Material and methods

### Experimental setup

The general experimental setup regarding the lysimeters (Figs. S1 and S2), the used soil and the amount of AgNM applied via sewage sludge in 2014 have been published in Schlich et al. (2017). In the following section, all methods are briefly described. The focus in the current study was on the second application of AgNM via sewage sludge to the existing lysimeters and the preparation of new lysimeters with AgNO_3_ or controls with pure sewage sludge without AgNM or AgNO_3_ application.

#### Soil

The experiments were conducted using a reference soil (RefeSol 01A, pH_CaCl2_ = 5.61, C_org_ = 9.3 g/kg, clay content = 61 g/kg), which was classified as Cambisol (Ad–hoc–AG Boden 2006). For detailed physicochemical properties, refer to Table S1. The soil was selected as reference soils by the German Federal Environment Agency, and it matches the properties stated in various OECD terrestrial ecotoxicological guidelines (e.g., tests with plants and soil microflora).

#### Silver nanomaterial and silver nitrate

A colloidal silver dispersion (NM-300K, rent-a-scientist GmbH, Regensburg, Germany), with a nominal silver content of 10% (w/w) and a transmission electron microscope diameter smaller than 20 nm, was used as AgNM. The particles are dispersed in a mixture of stabilizing agents (NM-300K DIS, rent-a-scientist GmbH, Regensburg, Germany) comprising 4% (w/w) each of polyoxyethylene glycerol trioleate and polyoxyethylene sorbitan monolaurate according to Klein et al. (2011). Silver nitrate from Merck (Merck KGaA, Darmstadt, Germany) was used as a pure material in the crystalline structure.

#### Sewage sludge

Sewage sludge, fed with municipal sewage, was freshly gathered at the WWTP of Schmallenberg (Germany). The sewage sludge met the requirements of the German Sewage Sludge Ordinance (AbfKlärV 1992) regarding the metal content (lead, cadmium, chromium, copper, nickel, mercury, zinc) of sewage sludge used as fertilizer on agricultural land. For the application of AgNM and AgNO_3_, sewage sludge was sieved to particles smaller than 2 mm and then stored in a vessel under permanent moderate stirring and aeration (2.5 mg O_2_/L).

AgNM and AgNO_3_ were spiked into sewage sludge by a ratio dry matter of sewage sludge sufficient to receive the desired nominal concentration after application via sewage sludge. After the addition of either AgNM or AgNO_3_ into sewage sludge, the sludge remained in the vessel for another 16 h under aeration and moderate stirring to allow transformation reactions of the AgNM or AgNO_3_ with the surrounding media and the interaction with the sewage sludge. To separate water and sewage sludge, the same flocculant as 2014 (0.2% cationic polyacrylamide solution, Sedifloc 154, Kemira Germany GmbH, Frankfurt, Germany) was applied.

#### Setup of lysimeters

The experiment is an extension of the lysimeter experiment described by Schlich et al. (2017). In the first part of the experiment, AgNM was incorporated into soil via sewage sludge. Over a period of three years, both the effect of AgNM on soil microorganisms and their fate in the uppermost 40 cm of the lysimeter and in leachate were investigated. In the second part of the experiment, sewage sludge (with and without AgNM) was again applied to the already existing lysimeters to investigate the influence of repeated AgNM input via sewage sludge on the fate and effect of AgNM. In addition, the fate and effect of AgNO_3_ in a lysimeter experiment was considered in comparison to AgNM.

Control lysimeters (unspiked sewage sludge, L1, L27) and lysimeters with concentrations of 2 mg AgNM/kg dry matter soil (DMS, L2) and 8 mg AgNM/kg DMS (L6) were tested in the first part of the experiment (Schlich et al. 2017). Again, 1.67 g dry matter sewage sludge/kg DMS was applied to the lysimeters (L1, L2, L6) in May 2018. In L2, AgNM was mixed to achieve a concentration of 2 + 3 mg AgNM/kg DMS. Here, the aim was to investigate whether a comparable effect could be achieved as it was observed for lysimeter 6 (8 mg AgNM/kg DMS) in the first part of the experiment. In addition, pure sewage sludge was applied to L6 (8 + 0 mg AgNM/kg DMS) to observe whether the detected strong inhibition further increased over time or if it reached a steady state or even decreased. In addition, a new lysimeter experiment was initiated to obtain data on the comparability of effects observed due to AgNO_3_ and AgNM. For the control (L27), only sewage sludge was applied to the soil, and for L28, a concentration of 2 mg Ag/kg DMS was introduced into the soil by AgNO_3_-spiked sewage sludge.

The artificially filled lysimeters (0.9 × 0.9 × 0.9 m; ≈1 t DMS) are cubic shaped and made of high-grade stainless steel. The volume of approximately 1 m^3^ of the soil presents a homogeneous and integrating system. For microbial determinations, samples can be collected at distinct locations to consider potential inhomogeneity. Since the aim of the study was to investigate the effects of AgNM and AgNO_3_ applied via sewage sludge to soil on soil microorganisms, no lysimeter containing only soil was included. There was no effect on the soil microflora (AOB and microbial respiration) due to NM-300K DIS (Schlich et al. 2013), the dispersant of NM-300K. Therefore, no separate lysimeter containing only the dispersing agent has been conducted.

According to the German sewage sludge ordinance (AbfKlärV 1992), sludge can be applied on agricultural land at an amount of 5 t/ha in three years. Following current practice, it was assumed that the complete amount of sewage sludge would be applied at once. In addition, a soil depth of 20 cm (in accordance with agricultural practice) and a soil bulk density of 1.5 g/cm³ (OECD Guideline 216 2000; OECD Guideline 217 2000) were assumed for the calculation of the amount of sewage sludge that could be applied to the soil.

Sewage sludge was applied as described by Schlich et al. (2017). Briefly, first sewage sludge was mixed over 30 min with 25 kg DMS, taken from the top layer (20 cm) of the lysimeters, to receive a homogeneous mixture of soil and sewage sludge containing either AgNM or AgNO_3_. Afterward, the mixture of soil-sewage sludge was spread on the top of the soil in the lysimeters and mixed into the top 20 cm with a spade. After the second application of sewage sludge to the lysimeters (L1 (control), L2, L6), the pH of the soil decreased (pH < 5). Therefore, in November 2018, 75 g of CaO (Quicklime for soil improvement; purity 82% (CaO + MgO), Zement- und Kalkwerke Otterbein GmbH & Co. KG, Großenlüder-Müs, Germany) was added to each lysimeter containing AgNM (L2, L6) and the control lysimeter (L1) to increase the pH values. No CaO was added to the new lysimeters (L27 (control), L28) since the pH was comparable to the pH observed in the AgNM lysimeters in 2014. After sewage sludge (with or without AgNM or AgNO_3_) was incorporated into the soil (May 2018), leachate was sampled, but there was no sampling of the soil to allow a new equilibrium to be established.

In June 2018, summer wheat (*Triticum aestivum* ´Tybalt A‘, Saaten Union GmbH Isernhagen, Germany) was sown followed by black fallow land from September 2018 to May 2019. The seeds were untreated for experimental purposes. Afterward, sugar beet (*Hordeum vulgare* ‘SY Typee’, Syngenta, Maintal, Germany) was sown in May 2019 and harvested in September 2019. Plants were observed on a weekly basis and irrigated with the same amount (10–30 L) of tap water in dry periods (L1, L2, L6, L27 and L28: July 18 until August 3rd, 2018).

At harvest of the wheat, approximately 50% of the plants were taken, including their roots. The remaining plants were cut 5 cm above the ground. The roots remained in the soil to prevent the removal of AgNM or AgNO_3_ accumulated in the roots. The harvested wheat plants were divided into roots and shoots before they were stored at −21 °C until analysis. The sugar beets were collected, and the soil was stripped away. The sugar beets were stored at 4 °C in a refrigerator until analysis. After harvesting, five soil samples per lysimeter were taken using a soil sampler (Pürkhauer drilling stick) for the top 40 cm and divided in steps of 10 cm.

The leachate of each lysimeter was permanently collected, and the volume was determined. Water samples were taken regularly, filtered (0.45 µm polyethersulfon syringe filter, VWR International GmbH, Langenfeld, Germany), and preserved with 100 µl of 69% HNO_3_ suprapur (Carl Roth GmbH + Co. KG, Karlsruhe, Germany) before analysis.

#### Ecotoxicological test system

The results of the first part of the experiment (Schlich et al. 2017) showed that AOB (potential ammonium oxidation, ISO Guideline 15685 2012) were more strongly inhibited by AgNM than the substrate induced respiration (SIR, OECD 217, 2000). For this reason, in the second part of the experiment, we focused on the effect of AgNM and AgNO_3_ on AOB. In accordance with ISO 15685 (ISO Guideline 15685 2012), the nitrite concentration was determined by a short-term potential ammonium oxidation test to observe the effects on soil nitrifying bacteria. The objective of ISO 15685 (2012) is to measure the ammonia oxidation potential, which provides an indication of the size of the ammonia oxidizing bacterial population.

#### Climate and soil conditions

The average monthly rainfall was between 40 and 199 mm, and the average monthly temperature was between −2.9 and 17.5 °C from June 2018 until October 2019. In L1, L2 and L6, the measured pH from June 2018 (addition of sewage sludge) until November 2018 (liming with CaO) was between 4.4 and 4.8 and increased to a maximum pH of between 6.1–6.6 in May 2019 (Table S2). In August 2019, the measured pH values were between 5.0 and 5.3. In the new lysimeters (L27 and L28) with a control and AgNO_3_, the pH was between 5.1 and 6.0 from June 2018 until May 2019 and decreased to a final pH from 4.9 to 5.0, which was comparable with L1, L2 and L6, until August 2018.

### Analytics

#### Soil, plant material, leachate

The soil pH was measured in 0.01 mol/L CaCl_2_ (Th. Geyer, Renningen, Germany) after 24 h of extraction using a inoLab pH 720 (WTW GmbH, Weilheim, Germany). According to DIN EN 16174:2012-11 (2012), aqua regia digestion (ARD) was applied to the ground soil samples. The Ag_total_ concentration after ARD was labeled as Ag_ARD_. According to Lowry et al. (2012), the dried and ground plant materials were digested with HNO_3_ (65%, Suprapur, Merck, Darmstadt, Germany). The volume of concentrated HNO_3_ was increased from 1.5 to 4 ml to enable a complete dissolution of the plant tissues. The Ag concentration after HNO_3_ digestion was labeled Ag_HNO3_. According to DIN 38402-11:2009-02 (2009), the leachates were filtered (0.45 µm, Graphic Controls, Buffallo, NY, USA) and acidified (69%, HNO_3_, Suprapur, Carl Roth GmbH & Co. KG, Karlsruhe, Germany) immediately after sampling. The measured Ag concentration of the leachates was labeled Ag_DIN38402_ Inductively coupled mass spectrometry (ICP-MS, 7700 Series, Agilent, Santa Clara, California, USA) and inductively coupled optical emission spectroscopy (ICP-OES, Ciros Vision, Spectro, Kleve, Germany) were used to determine the Ag concentration in the leachates and in the digested soil and plant materials.

### Statistics

Statistical analysis was conducted using ToxRatPro v3.3.0 software for ecotoxicity response analysis (ToxRat Solutions GmbH, Alsdorf, Germany) and SPSS 22.0.0.0 (IBM Corp., Armonk, USA). For the microbial tests, the Student’s *t* test for homogeneous variances (one sided, *p < 0.05; **p < 0.01; ***p < 0.001) was performed after proving homogeneity of variances tested by the Levene’s test (*α* = 0.05). In Fig. 4, significant *p* values are marked with asterisks.

The statistical analysis regarding the Ag_DIN38402_ concentration in the leachates of the lysimeters was executed with IBM SPSS Statistics. The measurement data were not normally distributed, which is why the non-parametric Mann–Whitney U test was applied. This test was used to assess whether the distributions of the Ag_DIN38402_ concentration from the AgNM-spiked and AgNO_3_-spiked lysimeters (L2, L6, L28) were equal to the control lysimeters (L1, L27), which is the null hypothesis. All deviations (±) of measurement data given in the text represent the standard deviations of at least three replicates.

## Results and discussion

### Silver concentration in the lysimeters soil

**Fig. 1:**
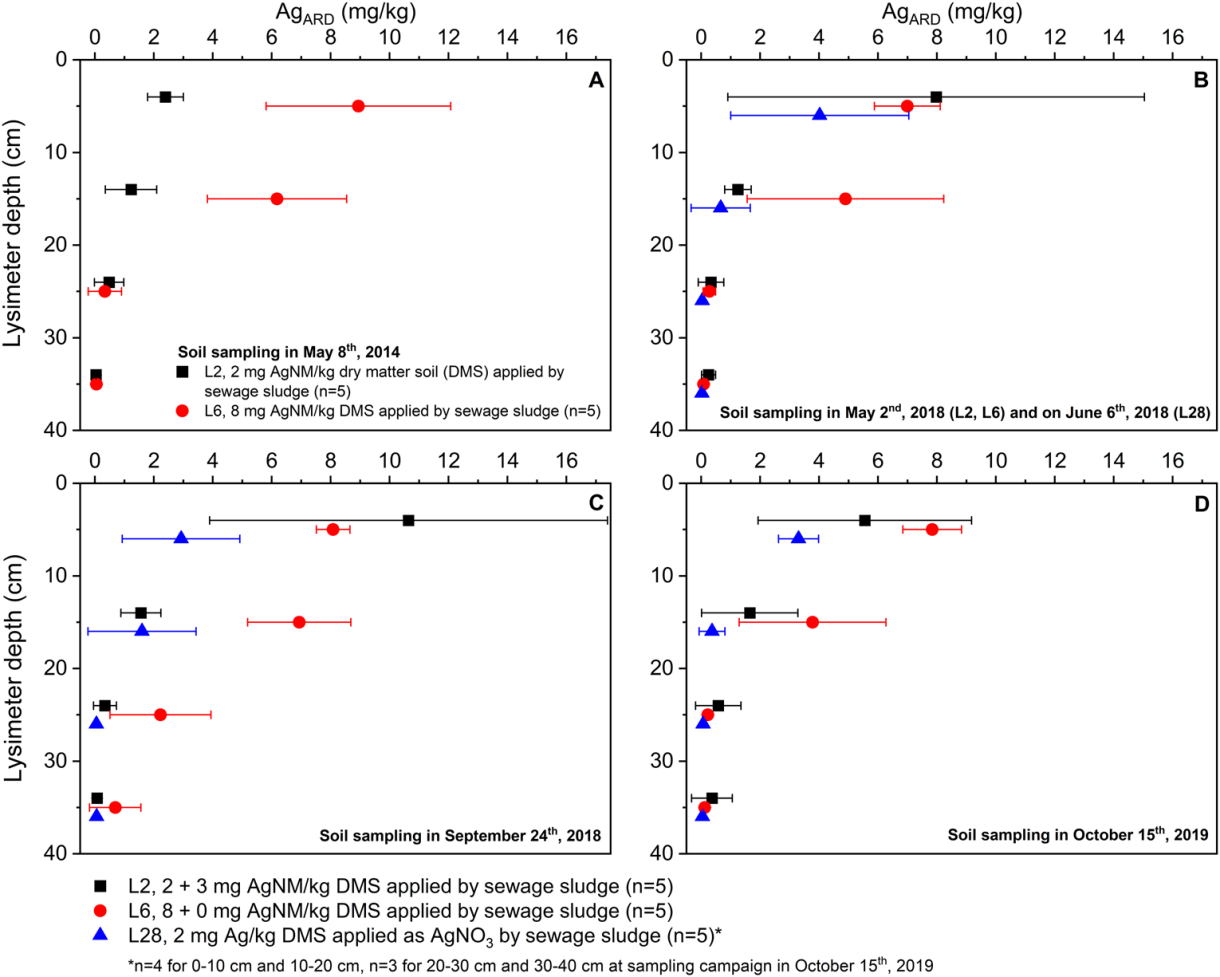
Total Ag concentration after aqua regia digestion (ARD) in the pooled samples of the four uppermost lysimeter layers (0–10, 10–20, 20–30, 30–40 cm). The silver nanomaterial (AgNM, OECD standard, NM-300K) was applied using sewage sludge to the two uppermost layers (0–10, 10–20 cm) in 2014 (lysimeter 2 (L2) and lysimeter 6 (L6)). In 2018, an additional application of AgNM (L2) and silver nitrate (AgNO_3_, lysimeter 28 (L28)) was conducted by sewage sludge. Error bars represent the standard deviation. Data in **A** are reprinted from Schlich et al. (2017). Control lysimeter 1 (L1) received pristine sewage sludge in 2014 and 2018, and control lysimeter 27 (L27) received pristine sewage sludge in 2018. The Ag concentration in the controls (data not shown) were below 0.05 mg Ag/kg dry matter soil (DMS) in all layers during the five years of the experiment

Figure 1A–D shows the timeline (May 8th, 2014; May 2nd, 2018; September 24th, 2018, October 15th, 2019) of the Ag_ARD_ concentration in pooled samples of the four uppermost lysimeter layers (0–10, 10–20, 20–30, and 30–40 cm). The Ag_ARD_ concentration was low in all control lysimeters (L1 and L27, max. Ag_ARD_ = 0.05 ± 0.04 mg/kg DMS, *n* = 5, data not shown). Therefore, the measured Ag_ARD_ in the other lysimeters can be assigned to the applied AgNM (L2 and L6) and to the applied AgNO_3_ (L28). The slightly enhanced Ag_ARD_ concentration in the third and fourth layers (Fig. 1C) of L6 can likely be explained by the inaccuracy of the sampling by the Pürckhauer drill. This explanation is supported by the fact that enhanced Ag_ARD_ concentration was not found in the third and fourth layers of L6 in the following sampling campaign (Fig. 1D).

Almost no vertical translocation of the applied AgNM was found during the five years of the long-term experiment, which is in line with other field lysimeter experiments that found high AgNM retention in the plowed Ap horizon with no transport to deeper soil horizons (Makselon et al. 2018). Moreover, the second application of pristine sewage sluge (L6, May 2nd, 2018) and AgNM-spiked sewage sludge (L2, May 2nd, 2018) induced no enhanced vertical translocation of the applied AgNM.

The application of AgNO_3_-spiked sewage sludge to L28 (June 6th, 2018) showed no translocation of sludge-applied Ag to deeper soil layers (Fig. 1B–D). Yang et al. (2020) found Ag migration in an outdoor lysimeter after application of AgNO_3_-spiked sewage sludge to a depth of 60–80 cm; however, most of the applied Ag also accumulated in the uppermost horizon. Please refer to the Sections below for further discussions about AgNM and AgNO_3_ retention mechanisms and comprehensive conclusions about the environmental fate of AgNM in soil.

The standard deviation of the Ag concentrations in the two uppermost soil horizons remained high during the five years of the experiments. This indicates that despite plowing, AgNM-spiked sludge remains in the area to which it was applied.

### Silver concentrations in the leachates

**Fig. 2:**
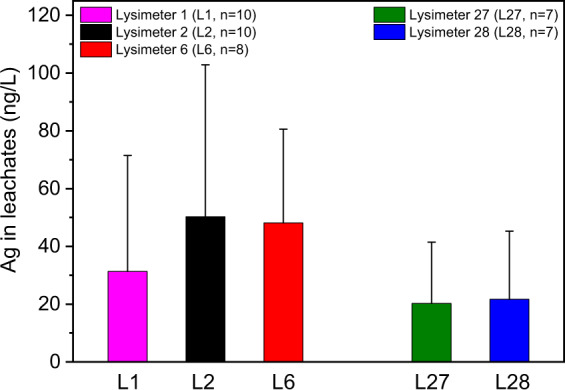
Silver concentration (Ag_DIN38402_) in the leachates of lysimeter 1 (L1, control, twofold application of unspiked sewage sludge), lysimeter 2 (L2, two applications of AgNM-spiked sewage sludge (2 + 3 mg AgNM/kg dry matter soil (DMS))), lysimeter 6 (L6, one application of AgNM-spiked and one application of unspiked sewage sludge (8 + 0 mg AgNM/kg DMS)), lysimeter 27 (L27, control, one application of unspikeded sewage sludge), lysimeter 28 (L28, one application of AgNO_3_-spiked sewage sludge (2 mg Ag/kg DMS)). All samples were sampled between May 18th, 2018 and May 13th, 2019. Error bars represent the standard deviation of ≥7 replicates. According to the Mann–Whitney U test null hypothesis (H_0_: The distribution of Ag concentration is equal between the control groups (L1, L27) and the Ag-spiked lysimeters (L2, L6, L28) was only declined for L1 vs. L2 (*p* < 0.05))

From May 2018 to May 2019 (Table S3), the leachates were sampled from the five lysimeters (*n* = 42) after rainfall events (Fig. 2). The highest release was at a low level (max. Ag_DIN38402_ = 176 ng/L, Lysimeter 2, November 28th, 2018) and was determined in a leachate of L2 with an AgNM concentration of 2 + 3 mg AgNM/kg DMS. The controls showed a very low Ag_DIN38402_ release with arithmetic means of 31 ng/L (L1, *n* = 10) and 20 ng/L (L27, *n* = 7). Lysimeter 2 obtained two applications, and L27 obtained one application of unspiked sewage sludge. Therefore, the higher release from L2 compared to L27 might represent the Ag background in the sewage sludge. According to the Mann–Whitney U test, the distribution of Ag_DIN38402_ concentrations released from the control (L1) and L2 (arithmetic mean 50 ng/L, *n* = 10) showed significant deviations (*p* = 0.043). A tendency of enhanced Ag concentrations compared to L1 (arithmetic mean 48 ng/L, *n* = 8) was also found for L6; however, deviations in the distribution of the Ag_DIN38402_ concentrations were not significant at the significance level of 0.05 (*p* = 0.067, Mann–Whitney U test). The low and continuous release of Ag to the leachates is comparable with the results of the previous study that investigated L1, L2 and L6 (Schlich et al. 2017). Other long-term field lysimeter studies are in line with these findings (Durenkamp et al. 2016; Makselon et al. 2018). Moreover, Makselon et al. (2018) showed that AgNM was associated with soil colloids in an undisturbed outdoor lysimeter. The presented Ag_DIN38402_ concentrations do not provide information about the Ag species. However, a preliminary column study with the same soil (Cambisol, Refesol 01 A) and AgNM showed that the low released concentration of Ag was associated with soil colloids (Hoppe et al. 2015), which is why the low measured Ag_DIN38402_ is probably associated with soil colloids. The applied concentrations of AgNM (L2 = 2 + 3 mg AgNM/kg DMS, L6 = 8 + 0 mg AgNM/kg DMS) led to a very low release of Ag_DIN38402_ (arithmetic mean ≤50 ng/L). The predicted environmental concentration in agricultural soils (<1 µg AgNM/kg DMS, Gottschalk et al. 2013) was far below the applied concentration in L2 and L6. Given that the experimental design with the used soil is transferable to real environmental conditions, a detectable release of AgNM from sewage sludge-treated agricultural soil to leachate and further to groundwater is unlikely.

The distribution of Ag_DIN38402_ concentrations released from the control (L 27 arithmetic mean 20 ng/L, *n* = 7) and the treatment with AgNO_3_ (L 28, arithmetic mean 22 ng/L, *n* = 7) showed no significant differences according to the Mann–Whitney U test (*p* = 1.00). Thus, the application of 2 mg Ag/kg DMS generated no release of Ag to the leachates. Additionally, Yang et al. (2020) found only a very low release of Ag to the leachates of an outdoor lysimeter after application of AgNO_3_-spiked sewage sludge. These findings are in line with batch experiments that showed a very high Ag adsorption capacity in 25 tested soil samples (Hoppe et al. 2014). Therefore, little or no risk of Ag release to leachates is predicted from Ag-containing sewage sludge applied to agricultural soil (≤2 mg Ag/kg DMS).

### Silver concentrations in roots of the crops

**Fig. 3:**
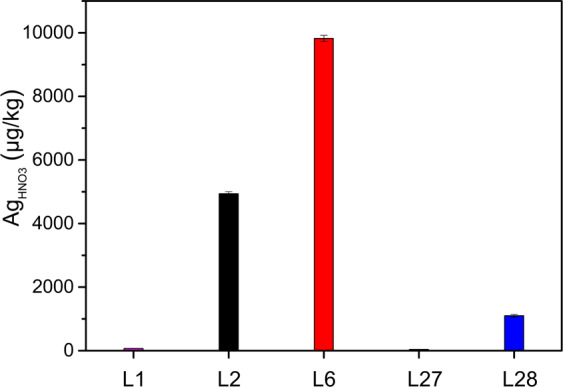
Silver (Ag_HNO3_) concentration in wheat roots (sampled on September 24th, 2018) from two control lysimeters (lysimeter 1 (L1), lysimeter 27 (L27)) with less than 0.05 mg Ag/kg dry matter soil (DMS), AgNM-spiked lysimeters (lysimeter 2 (L2), 2 + 3 mg AgNM/kg DMS, lysimeter 6 (L6), 8 + 0 mg AgNM/kg DMS), and AgNO_3_-spiked lysimeter (lysimeter 28 (L28), 2 mg Ag/kg DMS). Error bars represent the standard deviation of three replicates

Figure 3 shows enhanced Ag concentration in the AgNM- and AgNO_3_-spiked lysimeters (L2, L6, L28) in the wheat roots compared to the controls (L1, L27). The Ag concentration in the two uppermost layers of lysimeter soils (0–20 cm, September 24th, 2018) related to the Ag root concentration show only slightly enhanced transfer factors for the AgNM-spiked lysimeters (L2 = 0.8, L6 = 1.3) in contrast to the AgNO_3_-spiked lysimeter (L28 = 0.5). One can speculate that the roots grow to the areas where the sewage sludge is located to take up nutrients (e.g., nitrogen (N), phosphate (P), sulfur (S)). Silver nitrate was supposed to be sulfidized to Ag_2_S by sewage sludge before application to the soil of L28. Moreover, even bulk Ag_2_S was found to accumulate in oat roots (Schlich et al. 2018). Therefore, Ag uptake might be an unspecific comechanism induced by nutrient uptake, which might be related to the uptake of S.

**Fig. 4:**
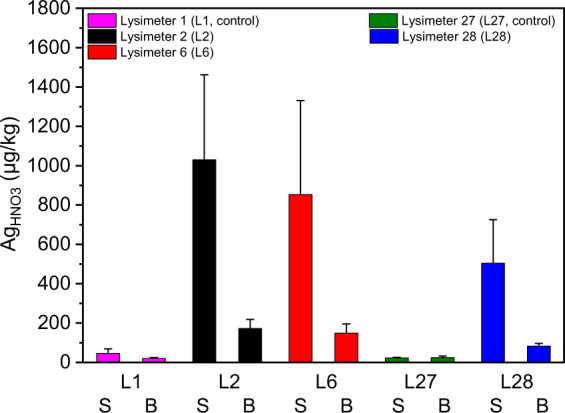
Silver (Ag_HNO3_) concentration in the skin (S) and body (B) of sugar beet roots (sampling September 24th, 2019). Lysimeters 1 (L1, two applications of unspiked sewage sludge) and lysimeter 27 (L27, one application of unspikeded sewage sludge) represent the controls (0.05 mg Ag/kg dry matter soil (DMS)), lysimeter 2 (L2, two applications of AgNM-spiked sewage sludge (2 + 3 mg AgNM/kg dry matter soil (DMS))), and lysimeter 6 (L6, one application of AgNM-spiked and one application of unspiked sewage sludge (8 + 0 mg AgNM/kg DMS)) represent the AgNM-spiked lysimeters, lysimeter 28 (L28, one application of AgNO_3_-spiked sewage sludge (2 mg Ag/kg DMS)) represents the AgNO_3_-spiked lysimeter. Error bars show the standard deviation of three replicates

The Ag wheat root accumulation in L6, which received only unspiked sewage sludge in 2018, was at the same level as the determined Ag wheat root concentration in 2014 (2018: 10 ± 0.1 mg Ag/kg dry matter plant (DMP) vs. 2014: 11 ± 0.8 mg Ag/kg DMP (data from Schlich et al. (2017)). This indicates that despite a second sludge application, the mobility of the AgNM applied in 2014 did not change until the end of the experiment in 2019. Therefore, the applied AgNM represents a low but continuous source for Ag uptake to roots, which suggests that AgNM can be transferred into the food chains up to top level consumers (Ruotolo et al. 2018). However, the transfer from the roots to stems was at a low level for wheat, with no detectable accumulation in the grains (Fig. S3). Reduced AgNM, AgNO_3_, and Ag_2_S-NM transfer through the root-shoot barrier was formerly found for alfalfa (*Medicago sativa* L.) by Stegemeier et al. (2015) and oat by Schlich et al. (2018). The root-shoot barrier might depict a limited risk for Ag species to enter the food chain under predicted environmental concentration in soil (<1 µg AgNM/kg DMS modeled by Gottschalk et al. (2013). Figure 4 shows increased transfer of Ag to the skin and body of sugar beets for the AgNM-spiked and AgNO_3_-spiked lysimeters compared to the controls. In addition, the comparable Ag beet root concentration in the AgNM-applied and AgNO_3_-applied lysimeters support the above discussed hypothesis that Ag uptake might be an unspecific comechanism induced by nutrient uptake. The higher Ag concentration at the root skin suggests that the Ag species might be retained on the surface of the roots. However, information about Ag species and Ag localization within plant roots cannot be provided due to low Ag root concentration (<1 mg/kg). Therefore, high-end techniques such as laser ablation (LA) ICP-MS, scanning electron microscopy (SEM), and synchrotron-based X-ray absorption spectroscopy (XAS) could not help to localize Ag species in root tissues. The mass-bound limitations of these techniques reflect a general problem investigating NM under environmentally relevant concentrations. Despite these limitations, the results indicate that the highest sugar beet root concentration (1.0 ± 0.4 mg Ag/kg DMP) are below the highest wheat root concentration (10 ± 0.1 mg Ag/kg DMP). Moreover, the maximal Ag concentration in the root bodies (0.17 ± 0.05 mg Ag/kg DMP) were below the maximal Ag concentration in the root skin (1.0 ± 0.4 mg Ag/kg DMP). Considering that the applied AgNM soil concentration (2–8 mg AgNM/kg DMS) are above the predicted AgNM soil concentration (<1 µg AgNM/kg DMS modeled by Gottschalk et al. (2013), the risk of AgNM entering the food chain via sugar beets might be low but cannot be excluded from the presented data.

### Inhibition of ammonium oxidizing bacteria by silver nanomaterial

Information on repeated applications of NM under environmentally relevant conditions is still sparse. In a previous study, we compared the effects of a single and repeated application of AgNM on soil microorganisms and found that after 28 d of testing, there was no significant difference (Schlich et al. 2016). However, for this study, a standardized test system was used, and AgNM was applied directly to the soil. The most relevant exposure pathway of NM into the environment will be via sewage sludge, which will be applied to agricultural land repeatedly over several years. In sewage sludge, the impact of NM and their effect on AOB can then be different. Therefore, we performed a second application of AgNM via sewage sludge to an already established outdoor lysimeter experiment (Schlich et al. 2017) to observe a potential increase in the previously observed inhibition of AOB by AgNM.

**Fig. 5:**
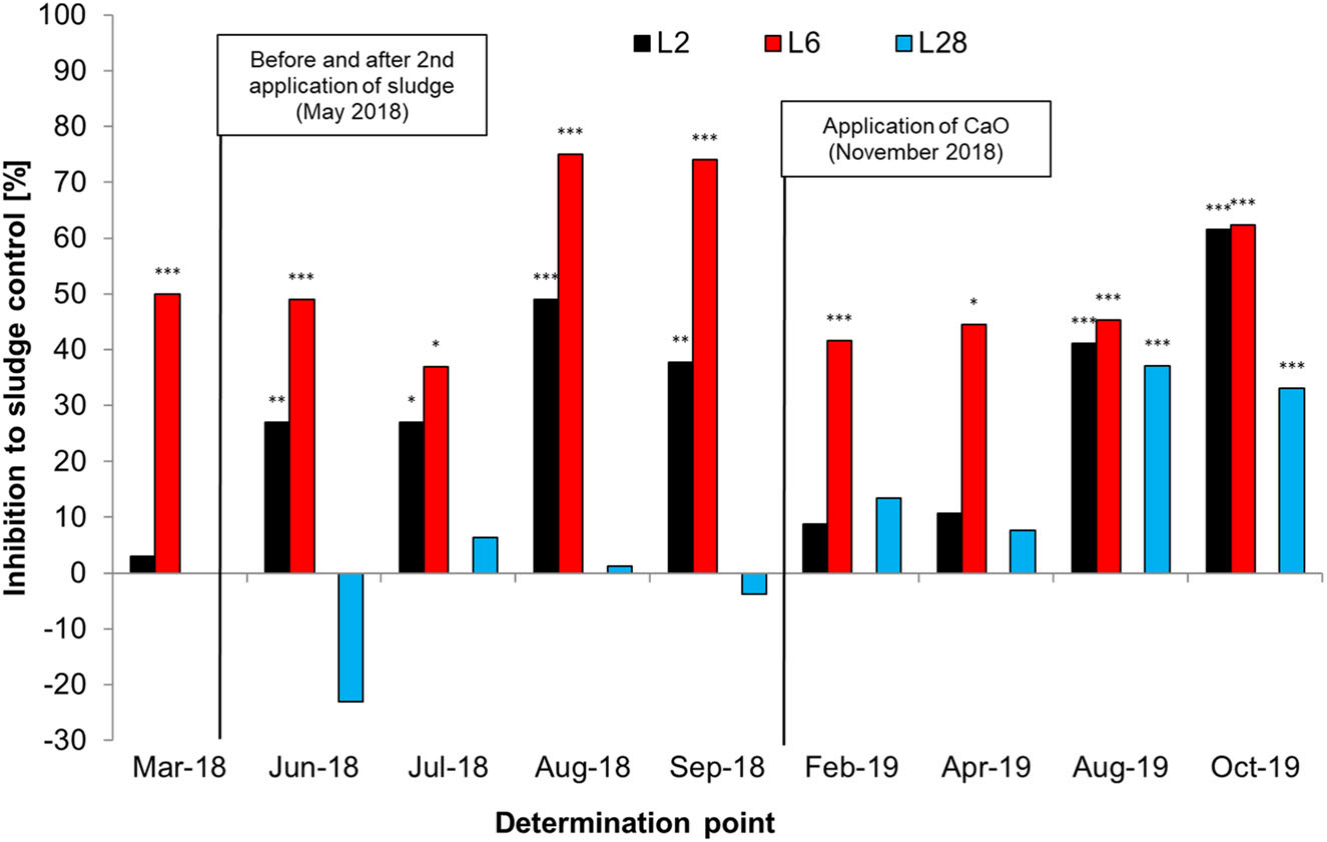
Inhibition of ammonium oxidizing bacteria due to AgNM (lysimeter 2 (L2), lysimeter 6 (L6) or AgNO_3_ (lysimeter 28 (L28)) applied to soil via sewage sludge over the complete course of the outdoor lysimeter study from March 2018 (before application) until October 2019 (end of experiment). In March 2018, L2 and L6 had measured concentration of 2 and 8 mg AgNM/kg dry matter soil (DMS). After the 2nd application was performed, the concentration in L2 was 2 + 3 mg AgNM/kg DMS, and 8 + 0 mg AgNM/kg DMS in L6. L28 had a concentration of 2 mg Ag/kg DMS. Statistics: Student’s *t* test, one sided, *p < 0.05; **p < 0.01; ***p < 0.001. For the absolute measured values and standard deviation, used for the statistical evaluation refer to Fig. S4

The second application of AgNM via sewage sludge on L2 was performed to increase the concentration from 2 mg AgNM/kg DMS to 2 + 3 mg AgNM/kg DMS. Three months after the 2nd application, there was an increase in the toxicity on AOB (Fig. 5). However, this increased toxicity was found in both lysimeters (L2 and L6), although only sewage sludge was added to the soil of L6. The inhibition of L2 increased from below 10% (March 2018 before the 2nd application) to 49% in August 2018, while the inhibition of AOB in L6 increased from 50 to 75% at the same time.

The results from Kraas et al. (2017) revealed that AgNM sulfidized under environmentally relevant conditions by passing a WWTP were still bioavailable to soil microorganisms over 140 d. In another long-term study over 90 d, it could be shown that processed AgNM, which had passed a WWTP, had a larger and more persistent effect on the microbial diversity and composition than freshly added AgNM, which was not processed within a WWTP (Forstner et al. 2020). In the experiment of Wang et al. (2016), AgNM applied via sludge to soil contained 87% Ag_2_S and 13% AgCl, whereas in soils amended with control sludge mixed with fresh AgNM or Ag_2_S-NM, most of the Ag was present as Ag_2_S. Wang et al. (2016) found that in general, 99% of AgNM was retained in sludge, and most of it (>79%) was transformed to Ag_2_S, which mainly remained in this state independent of various tested pH values and chloride concentration when incubated in soils for up to 400 d. Kraas et al. (2017) concluded that the transformed AgNM was partially amorphous, resulting in Ag_2_S species with, e.g., a higher solubility, differing from the solubility of crystalline Ag_2_S. Therefore, depending on the environmental conditions, an increased release of Ag^+^ from amorphous Ag_2_S species may explain the impact on AOB observed over 140 d (Kraas et al. 2017) and also in the present outdoor lysimeter experiment. The direct increase in the inhibition of AOB compared to the control (addition of sewage sludge only) after the 2^nd^ application in May 2018 was associated with a strong decrease in the soil pH (4.4–4.5 after application). The decrease in pH from approximately 5.3 (March 2018) to 4.5 could have caused a stronger release of ions from the amorphous Ag species, which then led to a stronger toxicity. The release of Ag^+^ in soil depends on the soil pH and has a strong influence on the toxicity to AOB, which was found for five different soils in a laboratory test (Schlich and Hund–Rinke 2015). However, linear regression analysis of the presented outdoor data also show a tendency of increasing AOB inhibition with decreasing soil pH, but the relation was not statistically significant. After the soil was limed with CaO (November 2018) and the pH increased continuously for several months up to pH 6.5 in May 2019, the inhibition of AOB concurrently decreased. As of August 2019, the pH was in the range of 5 to 5.3 in all lysimeters, resulting in an increase in the inhibition of AOB in L2.

The inhibition of AOB found at 2 + 3 mg AgNM/kg DMS in L2, to which a second amount of AgNM was added via sewage sludge, was comparable to the inhibition in L6, in which a concentration of 8 mg AgNM/kg DMS was tested since 2014. The inhibition in L6 did not fall below 40% over the entire experimental period. This indicates that the inhibition, which was determined in L6 since 2014, does not appear to be reversible. This, together with the low mobility of AgNM in soils, shows that repeated sewage sludge applications of sewage sludge containing AgNM can cause AgNM accumulation in the soil, leading to increasing inhibition of soil microorganisms.

### Inhibition of ammonium oxidizing bacteria by silver nitrate

After application of AgNO_3_ via sewage sludge to soil, there was initially no inhibition in the first few months from June to September 2018. The turnover of AOB in the control was comparable to the turnover measured at the test concentration (Fig. S4). After five months, an initial but statistically non-significant, inhibition of 13.5% was found (February 2019). When the pH decreased, an inhibition of 37% in August 2019 and 33% in October 2019 was detected. In comparison, this inhibition was between the effect of AgNM at concentration of 2 and 8 mg AgNM/kg DMS. This was probably again due to the variation in pH levels in the soil. Although the lysimeters in this part of the experiment were not limed in November 2018, these lysimeters were refilled in 2018, and the pH was already in the target range of 5.5 at the beginning of the experiment. However, afterward, the system needed some time to reach equilibrium, and then, as the experiment continued, the pH decreased slightly to 5.0 until the end of the test. In this phase, with a lower pH value, there was an increase in toxicity. Another reason for a delayed effect might also be the transformation of AgNO_3_ in sludge. Wang et al. (2016) found that AgNO_3_ that was processed in a WWTP or added to sewage sludge had transformed to Ag_2_S when it was amended to soil. After the soil was amended with AgNO_3_-spiked sludge, more than 92% of the total Ag in the sludge-amended soil was present as Ag_2_S after one day of incubation, suggesting that there was a fast transformation to Ag_2_S. In a study with soil only, it could be shown that when soils varying in pH were freshly spiked with AgNO_3_, acidic conditions favored the formation of AgCl, whereas Ag_2_S dominated in neutral or alkaline conditions (Sekine et al. 2015). Therefore, it might be possible that immediately after the transformation of AgNO_3_ to Ag_2_S, Ag was less toxic due to a reduced release of Ag^+^. However, with changing environmental conditions, a decrease in the pH, which likely accelerated the ion release, caused more pronounced toxic effects on AOB.

The presented results do not provide an exact explanation for the toxicity. In laboratory tests without sewage sludge, AgNO_3_ primarily showed short-term toxicity with direct effects after application (Schlich et al. 2013). However, AgNO_3_ showed a comparable effect to AgNM in this long-term outdoor lysimeter study. This suggests that a transformation to Ag_2_S, as found by several groups, also occurred for AgNO_3_, resulting in long-term toxicity.

### Overall discusion

The neglectable Ag transfer to the deeper soil layers and the neglectable transfer from soil to leachates indicate that the sludge-applied AgNM and AgNO_3_ remained nearly immobile in the soil. These findings are in line with the common state of knowledge that soil is a large sink for AgNM (e.g., Cornelis et al. 2014) and other NM (Hoppe et al. 2019). This study confirmed the state of knowledge for the first time over a period of five years and twofold sewage sludge application. The same AgNM and soil tested in this study were previously tested in short-term column experiments and showed a very low release of Ag associated with natural soil colloids (Hoppe et al. 2015). Despite different experimental designs (application, duration), the laboratory column experiments and outdoor lysimeter experiments led to the same conclusion that soils are a large sink for AgNM. In the case of sludge-applied AgNM and AgNO_3_, short-term laboratory experiments appeared to be sufficient to estimate the Ag release that occurred in the presented long-term field experiments. However, the application to sewage sludge and subsequent long-term incorporation into an outdoor lysimeter represents a more realistic environmental pathway and is mandatory to assess the uptake of Ag species to crops. Silver uptake from AgNM-spiked and AgNO_3_-spiked soils was determined for wheat and sugar beets. Previously, Stegemeier et al. (2015) conducted hydroponic exposure of alfalfa and indicated AgNM, Ag_2_S-NM, and AgNO_3_ accumulation along the apoplasts of the roots but only a low translocation to the shoot system. Root accumulation was also found for oat after exposure to different Ag species (AgNM, Ag_2_S-NM, AgNO_3_) spiked into soil by sewage sludge (Schlich et al. 2018). Root uptake suggests that AgNM can be transferred into food chains up to top-level consumers (Ruotolo et al. 2018). This assumption is not falsifiable from the presented findings, although the measured uptake from AgNM-spiked and AgNO_3_-spiked lysimeters was at a low level, and only limited transfer through the root-shoot barrier was identified.

Spurgeon et al. (2020) addressed the urgent need for long-term studies on the toxicity of NM. The inhibition of AOB and the high retention of AgNM applied to soil via sewage sludge led to increasing inhibition of soil microorganisms. Even though the investigated concentration up to 8 mg AgNM/kg DMS seem very high in relation to predicted environmental concentration, repeated application of sewage sludge over a long period of time, even when considering the presence of other NM (mixed toxicity), may lead to an adverse effect on soil microorganisms. Grün and Emmerling (2018) observed an effect on autotrophic ammonia oxidation (nitrification) and organic carbon transformation in soils at a very low AgNM concentration of 0.01 mg AgNM/kg DMS. Taking into account that the predicted environmental concentration (<1 µg AgNM/kg DMS modeled by Gottschalk et al. (2013)) are far below the applied AgNM concentration in this study, the potential risk of AgNM entering the groundwater or affecting AOB is nearly negligible. However, a risk for the food chain cannot completely be excluded from the presented data because of the measured AgNM and AgNO_3_ plant uptake. The localization and speciation of AgNM in the plant tissue was not possible because of the lack of analytical techniques to determine NM under predicted environmental concentration (Nowack et al. 2015). To further the debate on whether NM might compromise the food chain, further technical developments are necessary.

## Conclusion

Almost no vertical translocation or release of AgNM and AgNO_3_ to the percolating water was detected, even though the applied concentration was fare above the predicted environmental concentration. The second application of AgNM-spiked and pure sewage sludge to the existing lysimeters showed no enhanced remobilization of AgNM. Therefore, agricultural soils are a large sink for AgNM and AgNO_3_ applied by sewage sludge. However, AgNM and AgNO_3_ application resulted in comparable inhibition of AOB during the entire field lysimeter experiment. Moreover, an increasing inhibition of AOB was identified with repeated application via sewage sludge, which clearly indicates an accumulation effect. Due to the repeated application of sludge over many years, the soil microbial community might be exposed to a high risk.

In summary, the results indicate that AgNM and AgNO_3_ will partly be remobilized in the rhizosphere and translocated to the roots of wheat and sugar beet. Hence, AgNM will be bioavailable (AOB, crops) over several years, although root accumulation is low. However, the applied AgNM concentration was above the predicted environmental concentration, which explains why the observed root uptake might be neglectable in agricultural practice.

## Supplementary information

Supplementary Information

## References

[CR1] AbfKlärV (1992) Deutsche Klärschlammverordnung, 15. April 1992 (BGBl. I S. 912). https://www.bgbl.de/xaver/bgbl/start.xav?start=//*%5B@attr_id=%27bgbl192s0912.pdf%27%5D#__bgbl__%2F%2F*%5B%40attr_id%3D%27bgbl192s0912.pdf%27%5D__1629735325928

[CR2] Ad-hoc-AG Boden (2006) Bodenkundliche Kartieranleitung. 5. verbesserte und erweiterte Auflage. BGR in Zusammenarbeit mit den Staatlichen Geologischen Diensten (ed), (pp. 438): Schweizerbart Science Publishers, Stuttgart. https://www.schweizerbart.de/publications/detail/isbn/9783510959204/Bodenkundliche_Kartieranleitung_5_Aufl

[CR3] Benn T, Cavanagh B, Hristovski K, Posner JD, Westerhoff P (2010). The release of nanosilver from consumer products used in the home. J Environ Qual.

[CR4] Bundschuh M, Filser J, Lüderwald S (2018). Nanoparticles in the environment: where do we come from, where do we go to?. Environ Sci Eur.

[CR5] Cornelis G, Hund-Rinke K, Kuhlbusch T, van den Brink N, Nickel C (2014). Fate and bioavailability of engineered nanoparticles in soils: a review. Crit Rev Environ Sci Technol.

[CR6] Courtois P, Rorat A, Lemiere S, Guyoneaud R, Attard E, Levard C, Vandenbulcke F (2019). Ecotoxicology of silver nanoparticles and their derivatives introduced in soil with or without sewage sludge: a review of effects on microorganisms, plants and animals. Environ Pollut.

[CR7] DIN 38402-11:2009-02 (2009) German standard methods for the examination of water, waste water and sludge—general information (group A)—Part 11: sampling of waste water (A 11). Beuth Verlag, Berlin. https://www.beuth.de/de/norm/din-38402-11/108855969

[CR8] DIN EN 16174 (2012) Sludge, treated biowaste and soil—digestion of aqua regia soluble fractions of elements; German version EN 16174: 2012. Beuth Verlag, Berlin. https://www.beuth.de/de/norm/din-en-16174/148093733

[CR9] Durenkamp M, Pawlett M, Ritz K, Harris JA, Neal AL, McGrath SP (2016). Nanoparticles within {WWTP} sludges have minimal impact on leachate quality and soil microbial community structure and function. Environ Pollut.

[CR10] Forstner C, Orton TG, Wang P, Kopittke PM, Dennis PG (2020). Wastewater treatment processing of silver nanoparticles strongly influences their effects on soil microbial diversity. Environ Sci Technol.

[CR11] Gardea-Torresdey JL, Rico CM, White JC (2014). Trophic transfer, transformation, and impact of engineered nanomaterials in terrestrial environments. Environ Sci Technol.

[CR12] Gottschalk F, Sun T, Nowack B (2013). Environmental concentrations of engineered nanomaterials: review of modeling and analytical studies. Environ Pollut.

[CR13] Grün AL, Emmerling C (2018). Long-term effects of environmentally relevant concentrations of silver nanoparticles on major soil bacterial phyla of a loamy soil. Environ Sci Eur.

[CR14] Hänsch M, Emmerling C (2010). Effects of silver nanoparticles on the microbiota and enzyme activity in soil. J Plant Nutr Soil Sci.

[CR15] Hoppe M, Mikutta R, Utermann J, Duijnisveld W, Guggenberger G (2014). Retention of sterically and electrosterically stabilized silver nanoparticles in soils. Environ Sci Technol.

[CR16] Hoppe M, Mikutta R, Utermann J, Duijnisveld W, Kaufhold S, Stange CF, Guggenberger G (2015) Remobilization of sterically stabilized silver nanoparticles from farmland soils determined by column leaching. Eur J Soil Sci 66. 10.1111/ejss.12270

[CR17] Hoppe M, Schlich K, Wielinski J, Köser J, Rückamp D, Kaegi R, Hund-Rinke K (2019). Long-term outdoor lysimeter study with cerium dioxide nanomaterial. NanoImpact.

[CR18] ISO Guideline 15685 (2012) Soil quality—determination of potential nitrification and inhibition of nitrification—rapid test by ammonium oxidation. In: International Organization for Standardization (ed), Geneva, Switzerland. https://www.iso.org/standard/53530.html

[CR19] Kaegi R, Voegelin A, Ort C (2013). Fate and transformation of silver nanoparticles in urban wastewater systems. Water Res.

[CR20] Klein CL, Comero S, Stahlmecke J et al (2011) NM-Series of representative manufactured nanomaterials NM-300 Silver characterisation, stability, homogeneity. Tech. rep., JRC. 10.2788/23079

[CR21] Kraas M, Schlich K, Knopf B, Wege F, Kägi R, Terytze K, Hund-Rinke K (2017). Long-term effects of sulfidized silver nanoparticles in sewage sludge on soil microflora. Environ Toxicol Chem.

[CR22] Levard C, Hotze EM, Lowry GV, Brown GE (2012). Environmental transformations of silver nanoparticles: impact on stability and toxicity. Environ Sci Technol.

[CR23] Lowry G, Espinasse BP, Badireddy AR (2012). Long-term transformation and fate of manufactured Ag nanoparticles in a simulated large scale freshwater emergent wetland. Environ Sci Technol.

[CR24] Makselon J, Siebers N, Meier F, Vereecken H, Klumpp E (2018). Role of rain intensity and soil colloids in the retention of surfactant-stabilized silver nanoparticles in soil. Environ Pollut.

[CR25] Nowack B, Baalousha M, Bornhöft N (2015). Progress towards the validation of modeled environmental concentrations of engineered nanomaterials by analytical measurements. Environ Sci Nano.

[CR26] OECD Guideline 216 (2000). OECD guideline for the testing of chemicals.

[CR27] OECD Guideline 217 (2000). OECD guideline for the testing of chemicals.

[CR28] Pan B, Xing B (2012). Applications and implications of manufactured nanoparticles in soils: a review. Eur J Soil Sci.

[CR29] Ruotolo R, Maestri E, Pagano L, Marmiroli M, White JC, Marmiroli N (2018). Plant response to metal-containing engineered nanomaterials: an omics-based perspective. Environ Sci Technol.

[CR30] Schlich K, Klawonn T, Terytze K, Hund-Rinke K (2013). Hazard assessment of a silver nanoparticle in soil applied via sewage sludge. Environ Sci Eur.

[CR31] Schlich K, Hund-Rinke K (2015). Influence of soil properties on the effect of silver nanomaterials on microbial activity in five soils. Environ Pollut.

[CR32] Schlich K, Beule L, Hund-Rinke K (2016). Single versus repeated applications of CuO and Ag nanomaterials and their effect on soil microflora. Environ Pollut.

[CR33] Schlich K, Hoppe M, Kraas M, Fries E, Hund-Rinke K (2017) Ecotoxicity and fate of a silver nanomaterial in an outdoor lysimeter study. Ecotoxicology. 10.1007/s10646-017-1805-4 10.1007/s10646-017-1805-4 PMC5496968 28547324

[CR34] Schlich K, Hoppe M, Kraas M, Schubert J, Chanana M, Hund-Rinke K (2018). Long-term effects of three different silver sulfide nanomaterials, silver nitrate and bulk silver sulfide on soil microorganisms and plants. Environ Pollut.

[CR35] Sekine R, Brunetti G, Donner E (2015). Speciation and lability of Ag-, AgCl-, and Ag2S-nanoparticles in soil determined by X-ray absorption spectroscopy and diffusive gradients in thin films. Environ Sci Technol.

[CR36] Spurgeon DJ, Lahive E, Schultz CL (2020). Nanomaterial transformations in the environment: effects of changing exposure forms on bioaccumulation and toxicity. Small.

[CR37] Stegemeier JP, Schwab F, Colman BP et al. (2015) Speciation matters: bioavailability of silver and silver sulfide nanoparticles to Alfalfa (Medicago sativa). Environ Sci Technol 49. 10.1021/acs.est.5b01147 10.1021/acs.est.5b01147 26106801

[CR38] Tourinho PS, van Gestel CA, Lofts S, Svendsen C, Soares AM, Loureiro S (2012). Metal-based nanoparticles in soil: fate, behavior, and effects on soil invertebrates. Environ Toxicol Chem.

[CR39] Wang P, Menzies NM, Dennis PG (2016). Silver nanoparticles entering soils via the wastewater-sludge-soil pathway pose low risk to plants but elevated Cl concentrations increase Ag bioavailability. Environ Sci Technol.

[CR40] Wang P, Lombi E, Menzies NW, Zhao FJ, Kopittke PM (2018). Engineered silver nanoparticles in terrestrial environments: a meta-analysis shows that the overall environmental risk is small. Environ Sci Nano.

[CR41] Westerhoff P, Nowack B (2013). Searching for global descriptors of engineered nanomaterial fate and transport in the environment. Acc Chem Res.

[CR42] Yang L, Li S, Wu L, Ma Y, Christie P, Luo Y (2020). A field study of the fate of biosolid-borne silver in the soil-crop system. Environ Pollut.

